# Bulletin of Spanish Influenza

**Published:** 1918-10

**Authors:** 


### TEXAS MEDICAL JOURNAL

DR. F. E. DANIEL, Founder.

Established July, 1885

MRS. F. E. DANIEL, Publisher and Managing Editor.

VOL. XXXIV No. 4
AUSTIN, OCTOBER, 1918.

PUBLISHED MONTHLY.
Subscription:
$1.50

The publisher is not responsible for views of contributors.

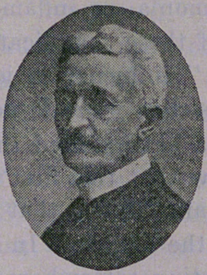

ASSISTANT EDITORS:
DR. JOHN S. TURNER, Dallas, Texas. DR. JOE BECTON, Greenville, Texas.
DR. E. B. PARSONS, Palestine, Texas. DR. M. F. BLEDSOE, Port Arthur.
DR. A. P. HOWARD, Houston, Texas. DR. J. H. FLORENCE, Dallas, Texas.
DR. J. H. REUSS, Cuero, Texas.

ARMY MEDICAL SEP 23 1947 LIBRARY

*"All of good the past hath had,*
*Remains to make our own time glad."*—WHITTIER.

### EDITORIAL.

### BULLETIN OF SPANISH INFLUENZA.

The Surgeon General of the U. S. Public Health Service has just issued the following publication dealing with Spanish Influenza, which contains all known available information regarding this diease. Simple methods relative to its prevention, manner of spread, and care of patients, are also given. Readers may obtain copies of this pamphlet free of charge by writing to the "Surgeon General, U. S. Public Health Service, Washington, D. C:"

### What Is Spanish Influenza? Is it Something New? Does It Come from Spain?

The disease now occurring in this country and called "Spanish Influenza" resembles a very contagious kind of "cold" accompanied by fever, pains in the head, eyes, back or other parts of the body, and a feeling of severe sickness. In most of the cases the symptoms disappear after three or four days, the patient then rapidly recovering; some of the patients, however, develop pneumonia, or inflammation of the ear, or meningitis, and many of these complicated cases die. Whether this so-called "Spanish" influenza is identical with the epidemics of influenza of earlier years is not yet known.

Epidemics of influenza have visited this country since 1647. It is interesting to know that this first epidemic was brought here from Valencia, Spain. Since that time there have been numerous epidemics of the disease. In 1889 and 1890 an epidemic of influenza, starting somewhere in the Orient, spread first in Russia, and thence over practically the entire civilized world. Three years later there was another flare-up of the disease. Both times the epidemic spread widely over the United States.

Although the present epidemic is called "Spanish influenza," there is no reason to believe that it originated in Spain. Some writers who have studied the question believe that the epidemic came from the Orient and they call attention to the fact that the Germans mention the disease as occurring along the eastern front in the summer and fall of 1917.

**How Can "Spanish Influenza" Be Recognized?**

There is as yet no certain way in which a single case of "Spanish influenza" can be recognized; on the other hand, recognition is easy where there is a group of cases. In contrast to the outbreaks of ordinary coughs and colds, which usually occur in the cold months, epidemics of influenza may occur at any season of the year, thus the present epidemic raged most intensely in Europe in May, June and July. Moreover, in the case of ordinary colds, the general symptoms (fever, pain, depression) are by no means as severe or as sudden in their onset as they are in influenza. Finally, ordinary colds do not spread through the community so rapidly or so extensively as does influenza.

In most cases a person taken sick with influenza feels sick rather suddenly. He feels weak, has pains in the eyes, head or back, and may be sore all over. Many patients feel dizzy, some vomit. Most of the patients complain of feeling chilly, and with this comes a fever in which the temperature rises to 100 to 104. In most cases the pulse remains relatively slow.

In appearance one is struck by the fact that the patient looks sick. His eyes and the inner side of his eyelids may be slightly "bloodshot" or "congested," as the doctors say. There may be running from the nose, or there may be some cough. These signs of a cold may not be marked; nevetheless the patient looks and feels very sick.

In addition to the appearance and the symptoms as already described, examination of the patient's blood may aid the physician in recognizing "Spanish influenza," for it has been found that in this disease the number of white corpuscles shows little or no increase above the normal. It is possible that the laboratory investigations now being made through the National Research Council and the United States Hygienic Laboratory will furnish a more certain way in which individual cases of this disease can be recognized.

**What Is the Course of the Disease? Do People Die of It?**

Ordinarily, the fever lasts from three to four days and the patient recovers. But while the proportion of deaths in the present epidemic has generally been low, in some places the outbreak has been severe and deaths have been numerous. When death occurs it is usually the result of a complication.

**What Causes the Disease and How Is It Spread?**

Bacteriologists who have studied influenza epidemics in the past have found in many of the cases a very small rod-shaped germ called, after its discoverer, Pfeiffer's bacillus. In other cases of apparently the same kind of disease there were found pneumococci, the germs of lobar pneumonia. Still others have been caused by streptococci, and by other germs with long names.

No matter what particular kind of germ causes the epidemic, it is now believed that influenza is always spread from person to person, the germs being carried with the air along with the very small droplets of mucus, expelled by coughing or sneezing, forceful talking, and the like by one who already has the germs of the disease. They may also be carried about in the air in the form of dust coming from dried mucus, from coughing, sneezing, or from careless people who spit on the floor and on the sidewalk. As in most other catching diseases, a person who has only a mild attack of the disease himself may give a very severe attack to others.

**What Should be Done by Those Who Catch the Disease?**

It is very important that every person who becomes sick with influenza should go home at once and go to bed. This will help keep away dangerous complications and will, at the same time, keep the patient from scattering the disease far and wide. It is highly desirable that no one be allowed to sleep in the same room with the patient. In fact, no one but the nurse should be allowed in the room.

If there is cough and sputum or ruuning of the eyes and nose, care should be taken that all such discharges are collected on bits of gauze or rag or paper napkins and burned. If the patient complains of fever and headache, he should be given water to drink, a cold compress to the forehead, and a light sponge. Only such medicine should be given as is prescribed by the doctor. It is foolish to ask the druggist to prescribe and may be dangerous to take the so-called "safe, sure and harmless" remedies advertised by patent-medicine manufacturers. If the patient is so situated that he can be attended only by some one who must also look after others in the family, it is advisable that such attendant wear a wrapper, apron or gown over the ordinary house clothes while in the sick room, and slip this off when leaving to look after the others.

Nurses and attendants will do well to guard against breathing in dangerous disease germs by wearing a simple fold of gauze or mask while near the patient.

**Will a Person Who Has Had Influenza Before Catch the Disease Again?**

It is well known that an attack of measles or scarlet fever or smallpox usually protects a person against another attack of the same disease. This appears not to be true of "Spanish influenza." According to newspaper reports the King of Spain suffered an attack of influenza during the epidemic thirty years ago, and was again stricken during the recent outbreak in Spain.

**How Can One Guard Against Influenza?**

In guarding against disease of all kinds, it is important that the body be kept strong and able to fight off disease germs. This can be done by having a proper proportion of work, play and rest, by keeping the body well clothed and by eating sufficient wholesome and properly selected food. In connection with diet, it is well to remember that milk is one of the best all-around foods obtainable for adults as well as children. So far as a disease like influenza is concerned health authorities everywhere recognize the very close relation between its spread and overcrowded homes. While it is not always possible, especially in times like the present, to avoid such overcrowding, people consider the health danger and make every effort to reduce the home overcrowding to a minimum. The value of fresh air through open windows cannot be over-emphasized.

Where crowding is unavoidable, as in street cars, care should be taken to keep the face so turned as not to inhale directly the air breathed out by another person.

It is especially important to beware of the person who coughs or sneezes without covering his mouth and nose. It also follows that one should keep out of crowds and stuffy places as much as possible, keep homes, offices and workshops well aired, spend some time out of doors each day, walk to work if at all practicable—in short, make every possible effort to breathe as much pure air as possible.

**"Cover up each cough and sneeze,**
**If you don't you'll spread disease."**

RUPERT BLUE, Surgeon General.

